# Discovery of a Recursive Principle: An Artificial Grammar Investigation of Human Learning of a Counting Recursion Language

**DOI:** 10.3389/fpsyg.2016.00867

**Published:** 2016-06-08

**Authors:** Pyeong Whan Cho, Emily Szkudlarek, Whitney Tabor

**Affiliations:** ^1^Department of Psychology, University of ConnecticutStorrs, CT, USA; ^2^Haskins LaboratoriesNew Haven, CT, USA

**Keywords:** counting recursion, artificial grammar learning, center embeddings, sequence learning, context-free grammar, graded state machine

## Abstract

Learning is typically understood as a process in which the behavior of an organism is progressively shaped until it closely approximates a target form. It is easy to comprehend how a motor skill or a vocabulary can be progressively learned—in each case, one can conceptualize a series of intermediate steps which lead to the formation of a proficient behavior. With grammar, it is more difficult to think in these terms. For example, center embedding recursive structures seem to involve a complex interplay between multiple symbolic rules which have to be in place simultaneously for the system to work at all, so it is not obvious how the mechanism could gradually come into being. Here, we offer empirical evidence from a new artificial language (or “artificial grammar”) learning paradigm, Locus Prediction, that, despite the conceptual conundrum, recursion acquisition occurs gradually, at least for a simple formal language. In particular, we focus on a variant of the simplest recursive language, *a*^*n*^*b*^*n*^, and find evidence that (i) participants trained on two levels of structure (essentially *ab* and *aabb*) generalize to the next higher level (*aaabbb*) more readily than participants trained on one level of structure (*ab*) combined with a filler sentence; nevertheless, they do not generalize immediately; (ii) participants trained up to three levels (*ab*, *aabb*, *aaabbb*) generalize more readily to four levels than participants trained on two levels generalize to three; (iii) when we present the levels in succession, starting with the lower levels and including more and more of the higher levels, participants show evidence of transitioning between the levels gradually, exhibiting intermediate patterns of behavior on which they were not trained; (iv) the intermediate patterns of behavior are associated with perturbations of an attractor in the sense of dynamical systems theory. We argue that all of these behaviors indicate a theory of mental representation in which recursive systems lie on a continuum of grammar systems which are organized so that grammars that produce similar behaviors are near one another, and that people learning a recursive system are navigating progressively through the space of these grammars.

## 1. Introduction

*Recursion* refers to “a process that calls itself” (Pinker and Jackendoff, 2005) and provides a way to “make infinite use of finite means” (Von Humboldt, 1999; see also Chomsky, 1957). In natural language, it refers to morphological and syntactic patterns in which one phrase is embedded inside another of the same type (e.g., [NP PP _NP_] or [NP [V [Comp [NP VP _S_] _CP_] _VP_] _S_]). Recursion is found in almost all natural languages, although it is still a matter of debate whether it is language universal (Everett, 2005; Nevins et al., 2009).

*Center-embedding recursion* [as in (1) below] refers to the case in which a phrase is embedded in the middle of a phrase of the same type. To process center-embedding recursion structures like (1-a) and (1-b), the language system must keep track of each constituent that has been started and not finished, and in cases like (1-a), the order in which these have occurred. If one assumes that the recursion processing system is capable of processing center-embedding patterns to arbitrary levels of embedding, then an infinite state mechanism is necessary for recognizing or generating all and only the legal structures (Chomsky, 1956; Hopcroft and Ullman, 1979).

(1) a. [The book [that the man [who hired me _S_] wrote _S_] deals with politics. _S_] (Cowper, 1976, quoted in Lewis, 1996)
  b. [[anti [[anti-[missile ${}_{N^{\prime }}$] _Adj_] missile ${}_{N^{\prime }}$] _Adj_] missile ${}_{N^{\prime }}$]

Christiansen and Chater (1999) distinguish two subtypes of center-embedding recursion: mirror recursion and counting recursion (see also Chomsky, 1957). The processor of a *counting recursion* language (e.g., *a*^*n*^*b*^*n*^) must keep track of the number of symbols of a particular type in order to generate or recognize all and only the strings of the language^1^. The processor of a *mirror recursion* language (e.g., {*ab*, *xy*, *aabb*, *axyb*, *xaby*, *xxyy*, *aaabbb*, *aaxybbb*, …}) must keep track not only of the number of symbols of a particular class, but also of their order.

Much debate in the artificial language learning domain has centered around the question of whether animals or humans trained in a laboratory setting can learn a mirror recursive language, as opposed to a mere counting recursive language (Fitch and Hauser, 2004; Perruchet and Rey, 2005; Bahlmann et al., 2006, 2008; Friederici et al., 2006; Gentner et al., 2006; Corballis, 2007; De Vries et al., 2008; Van Heijningen et al., 2009; Lai and Poletiek, 2011, 2013). Prior studies suggest that human learners can learn at least counting recursion in the lab and, according to Lai and Poletiek (2011), can learn mirror recursion if they are exposed to lower levels of embedding earlier than higher levels of embedding.

In this study, we aim to elucidate the process of recursion learning. We therefore focus on counting recursion, with which it is relatively easy to get robust generalization behavior, suggesting abstraction to a recursive rule. We argue that new insight into the way the human mind reaches and encodes such abstraction can be achieved by looking in detail at the learning process. To this end, we introduce a novel experimental task called “Locus Prediction” which provides rich information on the learning process.

Sometimes counting is categorized as a “strategy” and dismissed as uninteresting (e.g., De Vries et al., 2008). We agree that our participants, who certainly all learned how to count long before they participated in our experiment, may, in some cases, have eventually recognized the utility of using their counting ability to succeed with our task. We claim that it is the process of getting to the point of that recognition that is worth examining in detail. Indeed, as we report below, the task is not trivial in that many participants fail to solve it, and these show a range of behaviors which suggest that their minds are developing toward a formulation of the counting principle before they actually reach it. We also find that the work we have done on this counting recursion case establishes a foundation for analyses of mirror recursion cases, which we have explored in separate experiments (Tabor et al., 2012a; Cho et al., 2014).

What kind of novel understanding can be derived from this close examination of the learning process? There are three main points: (1) people seem to be able to proceed through a series of discrete grammatical systems which more and more closely approximate the target recursive grammar; (2) in between these states, they appear to occupy a continuum of states that smoothly transition from one discrete state to the next; (3) although it is natural to conceptualize (2) in terms of probabilistic grammar mixture, this conceptualization involves a challenging teleological assumption: people command a grammar of the future before they have fully adopted it (they mix it with a grammar of the past to form the current, blended grammar state). A careful empirical examination of the intermediate states indicates that something additional is occurring: the system exhibits a stability phenomenon, such that errors on one word enhance the chance of errors on a subsequent word, with the enhancement decreasing as each successive word is processed. This phenomenon is reminiscent of attractors in nonlinear dynamical systems theory. Helpfully, this theory offers a possible avenue out of the teleology. We go over these arguments carefully in General Discussion.

### 1.1. Motivating dynamical systems models

By *dynamical systems*, we mean formal systems that are characterized in terms of how they change. Typically, they are expressed as systems of differential equations or iterated maps (Strogatz, 1994). Several language-learning connectionist networks of syntactic processing are iterated map dynamical systems (Elman, 1990, 1991; Tabor, 2003, 2011). For example, the Simple Recurrent Network (SRN) (Elman, 1990, 1991) consists of a layer of input units which feeds forward to a recurrently connected layer of hidden units; this, in turn, feeds forward to a layer of output units. In the context of language learning, the SRN is trained to receive a sequence of words one word at a time and predict the next word. A prominent feature of such systems, which differentiates them from other models of cognitive processing, is their employment of continuous parameter and state spaces.

In the continuous *parameter* spaces (e.g., the weight space in the SRN), the structure of the knowledge representation continuously transforms as the systems learn (Tabor et al., 2013a). Tabor (2003) explores such a model, called a *Fractal Learning Neural Network* of the learning of some mirror recursion languages. He finds that the model progresses through a series of stages: early on, it can only handle sentences with one level of embedding. As training processes, the system masters two levels, then three, etc. with the times of mastery for each level decreasing in such a way that the system handles a close approximation of the infinite-state language after finite time. Moreover, between the mastery of each level, the system makes subtle changes in its encoding which shift it gradually from one level to the next (see also Tabor et al., 2013a). Inspired by these observations, we designed the experiments below to encourage participants to progress through successive levels and we sought evidence that they exhibit intermediate behavior when they transition between the levels.

In their continuous *state* spaces (e.g., the activation space in the SRN), typical dynamical systems are organized around *attractors* (Tabor et al., 2013b). An attractor is a subset of the state space with the following properties: (1) if placed inside the subset, the system will remain at it forever; (2) if placed slightly off (or *perturbed*) from the subset within some positive radius, the system will return to the set over time. The majority of research on dynamical systems has focused on autonomous systems: the system is a map (alternatively, a set of differential equations) from the state space to itself; attractors are often found among the limiting behaviors of such systems as time goes to infinity. Here, we are interested in a non-autonomous case: the system is driven simultaneously by its internal parameters and an environmental input that has a particular, predictable form: the environment is a series of symbolic events, the occurrence of symbols from a context free language which have a particular statistical distribution. Also in this case, it appears that the system's behavior can be organized around an attractor-like structure—i.e., a set to which the system returns when perturbed, provided that the environment obeys the grammatical structure following the perturbation^2^.

For example, in Tabor et al. (2013b), we ran numerical simulations of an SRN, training it on sentences randomly drawn from a finite state language, L = {*ab*, *aabb*, *aaabbb*}. We found that, when the trained model was processing sentences randomly drawn from L (with an exponential distribution of sentence types as a function of length), the model consistently occupied a restricted portion of its state space which we refer to as the “grammatical manifold.” When the trained model was perturbed by adding some random noise to its state or by presenting an unexpected word, it visited states off of the grammatical manifold immediately after the perturbation but gradually returned to the manifold in the course of processing grammatical sentences. Motivated by this observation, we sought evidence that humans who learned a recursive artificial language would gradually return to grammatical behavior after perturbation as they continued to receive grammatical input from the language. This prediction also hinges on the assumption that the system's states lie in at least an approximate continuum, because there need to be intermediate locations that it can occupy, as it returns gradually from the perturbed location back to the original trajectory.

### 1.2. Research hypothesis

We hypothesize that people trained on a sample of sentences from a recursive artificial language are gradually adjusting the continuous parameters of a dynamical system which is capable of developing attractor-like structures; this system is a highly plastic learning device and it can mirror the distribution of the input well. However, when the input exhibits sufficiently strong evidence for recursion, the system will generalize from the input to an approximation of the recursion. We made four predictions based on these assumptions and on our previous work with the Locus Prediction paradigm (Cho et al., 2011): (H1) training on low embedding levels of a recursive pattern should facilitate generalization to a higher level more than training on a non-recursive pattern, though the facilitation may not be immediate; (H2) training on more levels of recursive embedding should facilitate recursive generalization more than training on fewer levels of recursive embedding; (H3) a system learning a recursive pattern will proceed through stages^3^ corresponding to a series of grammars with successively deeper levels of embedding, and it will show gradual transitioning between the stages; (H4) an established pattern of behavior will show properties of an attractor: given an established pattern of behavior, unexpected events should perturb the system away from the established pattern, but as the system processes additional words that occur in grammatical sequence, the deviation magnitude will gradually decrease.

Complex learning developments may involve abrupt transitions such that it is possible to say, at one point in time, that the system has one structural character and that, a short time later, it has a qualitatively different character. In such a case, it is still possible that the transitions consist of continuous, but relatively rapid, changes. The importance of discovering such continuity, if it turns out to exist, is that it is currently a mystery how a complex, interdependent system like a recursive grammar can come into being in learning. Discovering some intermediate stages could help shed light on this mystery.

There is a natural alternative to our hypothesis about the character of grammar learning, suggested by many models that approach learning as a probabilistic structure selection process. For example, Perfors et al. (2011) consider a sample of finite state and context free grammar models of English word sequencing and they use a Bayesian scheme to progressively update the model's hypothesis about which grammars are most likely to have generated the input as more and more sentence examples are sampled from a training corpus. This type of model can also plausibly predict (H1) through (H3), but it does not predict the existence of the attractor structure (H4). Nor does it provide a clear motivation for the structures that it must assume to predict (H3). We further discuss the contrast between these views in General Discussion.

To demonstrate the acquisition of infinite-state recursion, one would need to test an infinite number of cases of unbounded length. This is not empirically feasible, and indeed, the evidence from natural language experiments indicates that exact recursive behavior (at least with mirror recursive cases) breaks down quickly as the number of levels grows (Miller and Isard, 1964; see discussion in Christiansen and Chater, 1999). To our knowledge, most prior artificial grammar studies on humans (Fitch and Hauser, 2004; Perruchet and Rey, 2005; Bahlmann et al., 2006; Friederici et al., 2006; Bahlmann et al., 2008; De Vries et al., 2008) have trained participants up to a particular level, *k*, of recursive embedding and then tested on novel combinations that have less than or equal to *k* levels^4^. Lai and Poletiek (2011) is a notable exception, but their method involves interleaving deeply embedded test exemplars with the training stimuli, raising the concern that the participants may have learned the deep embeddings from the test stimuli. Since a minimal finite state grammar for levels 1 through *k* fails to generalize to *k*+1 levels, and since performance on recursive structures degrades with level (Miller and Isard, 1964), training on levels 1 through *k* and testing on *k*+1 is an efficient way to obtain evidence for recursive generalization. We take this approach in this paper. Motivated by the numbers typical in real natural language we explored *k* = 2 and *k* = 3 in the experments reported here.

### 1.3. Overview

The remainder of the paper is organized as follows: in The Locus Prediction Task, we introduce our new task. In Experiments 1 and 2, we describe experimental results that lend support to the first two research hypotheses (H1 and H2). In Gradual Recursive Generalization, we introduce new methods of analysis, suitable to our novel paradigm which help test the hypotheses concerning gradualness, and describe data supporting the last two research hypotheses (H3 and H4). In General Discussion, we conclude.

## 2. The locus prediction task

A main goal of the present study is to understand developmental change in grammar during recursion learning. For this purpose, we need to efficiently obtain precise information about the structure of grammars learned by participants. Almost all the prior studies of recursion learning have employed the standard artificial grammar learning (AGL) paradigm (Reber, 1967; Saffran et al., 1996; for an exception, see Alexandre, 2010). Typically, participants are exposed to a set of grammatical sentences in the training phase and then are asked to judge if novel sentences are grammatical or not in the test phase. Although this approach provides an approximate analog of the real language learning situation, it has some shortcomings. First, it is inefficient because only one data point is collected per sentence. Second, there is a risk that people's knowledge is distorted by exposure to the ungrammatical examples that must be used in the test phase. Third, it is not feasible to glean detailed grammatical structure information during the process of training.

A widely employed method in sequence learning studies (for review, see Clegg et al., 1998), the *serial reaction time* (SRT) paradigm (Nissen and Bullemer, 1987; also see Cleeremans and McClelland, 1991; Alexandre, 2010), does give information about word-to-word transitions: participants observe a sequence of locations highlighted on a computer screen and they must click on each location after it is highlighted. Reaction times are recorded and used to infer information about what the participants have learned. However, even this method provides indirect and ambiguous information about the particular grammatical events participants are expecting at each juncture between words: a high response time on a particular word could occur (i) because the participant was expecting a different word (ii) because the word was expected, but difficult to process (cf. Gibson, 1998) (iii) because the word occurs at a point where it is natural to do some additional processing (for example, at the end of a phrase).

For the purpose of evaluating participants' knowledge more directly, the SRT task is often followed by a prediction task in which participants are given a sequence of locations (presented in the SRT task) and asked to predict a location to be highlighted (e.g., Cleeremans and McClelland, 1991; Jiménez et al., 1996). Alternatively, the SRT task can be followed by a generation task in which participants are asked to generate a sequence of locations following a given location (e.g., Destrebecqz and Cleeremans, 2001; Visser et al., 2007). Typically, the prediction or generation task is given once at the end of learning with the SRT task so does not allow the investigation of knowledge change during learning. A notable exception is Visser et al. (2007) in which the SRT task was alternated with the generation task to allow the evaluations of participants' knowledge at several time points. The use of prediction/generation with the SRT task is an excellent way of studying *implicit* sequence learning but it is not ideal when the research interest is in grammatical knowledge change: (i) Learning/training phases (with the SRT task) are strictly separated from evaluation/test phases (with the prediction/generation task) with an unrealistic assumption that participants do not learn during the evaluation phase. (ii) It is not easy to determine how to distribute the evaluation phases so that all important knowledge changes can be captured. (iii) It is not easy to detect fine-gained, possibly continuous change because inserting too many evaluation phases will hamper learning.

**Figure 1:**
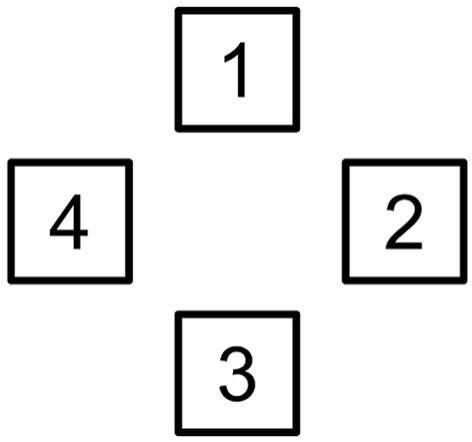
**A marked up initial display for the Locus Prediction task**. In the actual display, the boxes were not numbered.

We employed the Locus Prediction task (Cho et al., 2011; Tabor et al., 2012a,b) in which participants predict each successive symbol rather than react to it as in the SRT^5^. Participants initially observe a computer screen displaying an image like Figure 1 without the numbers. When they click on the screen, one of the boxes changes color. The color changes occur in accordance with a grammar: in this case, Grammar G_*R*_, shown in (2).

(2) S → 1 S 2 3 4
      S → 1 2 3 4

Grammar G_R_ generates a variant of the *a*^*n*^*b*^*n*^ language. Its sentences are [1 2 3 4] (level-1 sentence or S_1_), [1 [1 2 3 4] 2 3 4] (level-2 sentence or S_2_), [1 [1 [1 2 3 4] 2 3 4] 2 3 4] (level-3 sentence or S_3_), and so on. Sentences drawn from the grammar were sequenced by a pseudorandom process into one long symbol sequence of several hundred symbols' duration. Since the order of the sentences was essentially unpredictable, there was an indeterminacy whenever a 1 occurred: it might be followed by a 2 or it might be followed by another 1. We employed a coloring scheme to identify such nondeterministic events for participants: when a 1 occurred after another 1, the 1-box turned blue or cyan. On all other trials, one of the four boxes turned green. We told participants that they only needed to predict the green boxes (deterministic transitions). By only requiring participants to predict deterministic transitions, we made it possible, in principle, for them to perform perfectly on the task. When the same box had to change color on two trials in a row, the color was randomly chosen between blue and cyan under the constraint that the same color could not be used for two successive transitions. To reinforce the visual feedback that participants received when they failed to correctly predict a green box, a beep sounded^6^.

The Locus Prediction task has some advantages over the standard AGL task and the SRT task. First, Locus Prediction makes it possible to collect a response at every transition and, unlike in the SRT task, this response provides very specific information about how participants' understanding of the structure of the language evolves during the experiment. For example, Tabor et al. (2012b) studied progression of the grammatical system by investigating prediction behavior in windows across training. In the current study, we also take advantage of the rich information the task yields in order to detect behaviors that occur during the learning phase and seem to be mixtures of various simple grammatical systems. Second, no ungrammatical sentences are used so there is no risk of distorting a learner's developing knowledge with misleading ungrammatical information. Third, as we show below, accuracy rates in this task can be very high, providing strong evidence of recursive generalization. (We define *recursive generalization* as the significant tendency to generalize correctly to an untrained and deeper level of embedding than any of the levels experienced previously.) Fourth, human behaviors can be directly compared to the outputs of the Simple Recurrent Network (e.g., Cho et al., 2011). The SRN has been widely used to study sentence processing and offers a formally unified account of diverse sequence learning behaviors.

It is true that Locus Prediction involves explicit prediction of upcoming words, and thus does not resemble the most common mode of learning and using natural language, in which listeners do not explicitly predict upcoming words. There is now extensive evidence from the psycholinguistic literature that prediction is an integral part of word and sentence processing (Altmann and Kamide, 1999; Hale, 2001; Dahan and Tanenhaus, 2004; Levy, 2008). Also, as pointed out by Elman (1991), children might make covert predictions when they are learning; if this is right, then our task could be thought of as amplifying an action that children are engaging in anyway. By giving feedback on prediction at every step, we are able to watch some participants become very skilled predictors in the course of a 15–20 min experiment, shedding light, we think, on the emergence of this critical ability.

## 3. Experiment 1

In Experiment 1, we tested the hypothesis (H1) that training on low embedding levels facilitates recursive generalization to one deeper level of embedding more than training on a non-recursive pattern. Given a finite training set, participants can perform perfectly on the training set by memorizing each of its sentences (H0). If this is how they succeed on the training set, we expect generalization behavior to be equally poor, no matter what sentences they are trained on. If, on the other hand, the participants induce a recursive rule, we expect generalization to deeper levels of recursion. To compare H1 and H0, we created two finite languages, L_1_ and L_2_, both of which consisted of three sentences of different lengths (see Table 1). The first two sentences of L_1_ have a simple recursive relationship to one another in the sense that there is a context free grammar G_R_, such that the parse of S_1_ is a subtree of the parse of S_2_. On the other hand, the first two sentences of L_2_ do not have a simple recursive relationship in the sense that there is no context free grammar for which the parse of S1 is a subtree of the parse of S2. In Experiment 1, we exposed “Sequence 1” participants to the first two types from L_1_ and “Sequence 2” participants to the first two types of L_2_. We then probed the performance of the participants in each group on S_3_. We predicted that the participants exposed to Sequence 1 would perform better on S_3_ than the participants exposed to Sequence 2.

**Table 1:** **Finite languages used in Experiment 1 (L_1_ and L_2_) and 2 (L_3_)**.

**L_1_**	**L_2_**	**L_3_**
S_1_ = 1 2 3 4	S_1_ = 1 2 3 4	S_1_ = 1 2 3 4
S_2_ = 1 1 2 3 4 2 3 4	S${}_{2}^{*}$ = 1 1 4 3 2 4 2 3	S_2_ = 1 1 2 3 4 2 3 4
S_3_ = 1 1 1 2 3 4 2 3 4 2 3 4	S_3_ = 1 1 1 2 3 4 2 3 4 2 3 4	S_3_ = 1 1 1 2 3 4 2 3 4 2 3 4
		S_4_ = 1 1 1 1 2 3 4 2 3 4 2 3 4 2 3 4

### 3.1. Materials and methods

#### 3.1.1. Participants

Ninety-nine undergraduate students of the University of Connecticut participated in the experiment for course credit. This and the following experiments were approved by the University of Connecticut Institutional Review Board, and all participants provided informed consent.

#### 3.1.2. Materials and design

We used a between-subjects, single factor design in which the sequence of words presented to participants was manipulated across two levels (SeqType = Sequence 1 vs. Sequence 2). Fifty and forty-nine participants were assigned to the Sequence 1 and Sequence 2 conditions, respectively.

A sequence of 404 words (Sequence 1) was created by concatenating many instances of the three sentence types (S_1_, S_2_, and S_3_) ordered by a pseudorandom process and obeying the following constraints: First, the first 300 words (corresponding to the study phase) consisted of 31 S_1_s and 22 S_2_s. Second, S_2_s occurred less frequently at the beginning and more frequently at the end. Third, the last 104 words (corresponding to the test phase) consisted of 8 S_1_s, 3 S_2_s, and 4 S_3_s. The sequence ended with an S_1_. The actual sequences used in Experiment 1 and 2 are presented in Supplementary Materials, Section 1^7^. Because we did not collect participants' predictions at the last trial, we excluded their predictions on the last S_1_. Note that no S_3_s were presented before the beginning of the test phase. Thus, examining the test phase provides an opportunity to examine generalization from lower levels of embedding to higher levels. Note also: from the participants point of view, there was nothing marking the division between one sentence and the next nor was there any division between the training and test phases—the materials came as one long sequence of symbols.

Another sequence of 404 words (Sequence 2) was created by replacing S_2_s in Sequence 1 with ${\text{S}}_{2}^{*}$s (= 1 1 4 3 2 4 2 3); thus Sequence 2 had the same sentence-type distribution as Sequence 1. Given that there is no recursive relation between S_1_ and ${\text{S}}_{2}^{*}$, the experience of S_1_s and ${\text{S}}_{2}^{*}$s was not expected to help generalization to S_3_s in the test phase as in Sequence 1.

#### 3.1.3. Procedure

The experiment was run individually on a PC using E-Prime (Version 1.2) (Schneider et al., 2012). Participants were given a description of the locus prediction task (see Section The Locus Prediction Task) and then performed the task. The computer program recorded the responses and response times. Each session took about 15 min.

### 3.2. Results

One participant who was trained on Sequence 2 was excluded from the further analyses because the individual's mean accuracy across all trials was very low (*M* = 0.082); the participant did not predict which box would change color but clicked on whatever box had changed color after it changed color.

Sentence-level prediction accuracy, SentAcc, was defined as follows: SentAcc = 1 if all deterministic transitions of a level-*n* sentence were correctly predicted; SentAcc = 0, otherwise. By deterministic transitions, we mean the transitions that can be predicted with certainty. Let T_*ij*_ be a transition from box *i* to box *j*. In our simple language, T_11_ and T_12_ (plus T_14_ in Sequence 2) are nondeterministic transitions and all the other transitions are deterministic.

**Figure 2:**
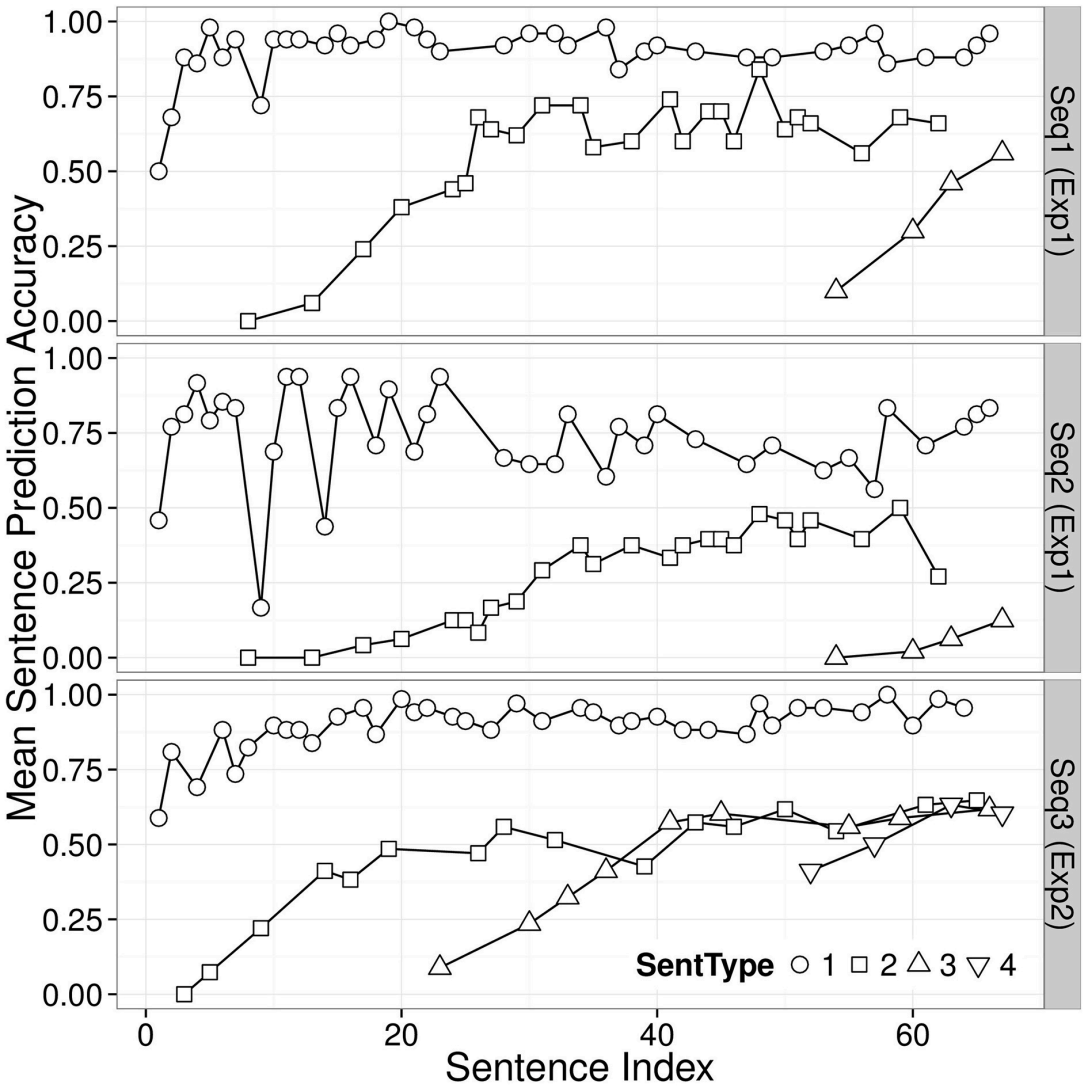
**Trajectories of average sentence prediction accuracy (SentAcc), separated by sentence types (SentType) in Experiment 1 (the top and middle panels), and Experiment 2 (the bottom panel)**. The mean sentence prediction accuracy represents the proportion of the participants who correctly processed all deterministic transitions of a level-*n* sentence at a particular position in a sequence of sentences.

The top and middle panels of Figure 2 show trajectories of the average sentence prediction accuracy (SentAcc) against the position of sentence in each sequence (Sentence Index) separated by sentence types (SentType) in Sequence 1 and Sequence 2 conditions^8^. The test phase started with the first instance of S_3_. In the Sequence 1 condition, SentAcc of S_3_ increased from very low level to reasonably high level with more experience of the sentence; in the Sequence 2 condition, SentAcc of S_3_ did not increase as much.

We asked: Is there any evidence that the participants in either condition generalized their experience from the training phase to the test phase?

First, we noted that five of 50 participants (10%) correctly processed the first instance of S_3_ in the Sequence 1 condition. By contrast none of the 48 participants in the Sequence 2 condition correctly processed the first instance of S_3_. This numerical contrast is in line with our prediction that Sequence 1 participants would generalize better, but it is not a significant result. A chi-squared test indicates that the two distributions are not significantly different, $\chi_{\left(1\right)}^{2}=3.20$, *p* = 0.073.

Because Locus Prediction gives us detailed information about the processing of each sentence, we can go further. We examined all the trials in the first instance of S_3_ for each participant. To be conservative, we assume that all participants in the Sequence 1 condition could correctly predict T_23_ and T_34_ transitions (recall that T_*ij*_ refers to a transition from Box i to Box j) because the transition probability was 1 in Sequence 1 and we ignored nondeterministic transitions because they cannot be predicted with certainty. Thus, we have three remaining transitions: two T_42_s and one T_41_ (associated with three positions [boldfaced] of S_3_ [1 1 1 2 3 **4** 2 3 **4** 2 3 **4**]). Note that the correct prediction of the next word upon receiving word 4 requires the knowledge of a nonadjacent dependency. Because only 1 and 2 occurred after 4 in the study phase, we assume that the learners can reject 3 and 4 regardless of whether they have acquired a recursive grammar. Thus, on each of the three remaining transitions, participants have a 50% chance of getting it right if they do not know the recursive pattern but are guessing randomly between boxes 1 and 2. This gives a chance level of 0.125 (= (1/2)^3^) for the three trials combined. The exact binomial test indicates that the proportion (= 0.1) of those who generalized is not different from chance (= 0.125), *M* = 0.1, 95% CI = [0.033, 0.218], *p* = 0.830. Thus, contrary to our expectation, we found no evidence that participants trained with Sequence 1 anticipated the recursive extension of their training experience. No one exposed to Sequence 2 achieved spontaneous generalization^9^.

So far, we have found no evidence for generalization in line with our prediction, but our hypothesis of a representational continuum makes a further prediction: Sequence 1 participants' representations may be close to recursive insight without actually being there, while we expect Sequence 2 participants' representations to be further away from recursive insight. Therefore, we predict that with the further training on S_3_'s provided by the Test Phase, Sequence 1 participants should quickly learn the more deeply embedded pattern, while Sequence 2 participants should be slower to do so.

We tested whether the sentence prediction accuracy on a novel sentence type S_3_ introduced in the test phase is different between two sequence conditions, using a loit mixed-effects model (Jaeger, 2008)^10^. The relative position of S_3_ (IndexS3 = 1, 2, 3, 4) and sequence type (SeqType) were included as fixed effects^11^. A by-subject random intercept and a by-subject random slope of IndexS3 were included as well without assuming a correlation between them^12^. The likelihood ratio tests between this model and another model with either one of the main effects excluded revealed that both main effects of IndexS3 and SeqType were statistically significant: *b*_IndexS3_ = 1.301, *SE*_IndexS3_ = 0.490, $\chi_{\left(1\right)}^{2}=4.44$, *p* = 0.035; *b*_SeqType_ = −5.855, *SE*_SeqType_ = 1.252, $\chi_{\left(1\right)}^{2}=17.20$, *p* < 0.0001. The odds ratio of SeqType (Seq2 to Seq1), 0.003 (= *e*^−5.855^), suggests that participants given Sequence 2 were much worse in processing S_3_s than participants given Sequence 1, although the sentences were equally new to both groups of participants.

It is true that there are more grammatical continuations for each word in Sequence 2 than in Sequence 1. For example, 4 is followed by 1 or 2 in Sequence 1 while it is followed by 1, 2, or 3 in Sequence 2. This means that, after the training phase, a bigram model predicts higher average prediction accuracy in Sequence 1 than in Sequence 2 as observed. However, the bigram model cannot explain the observed trial-level prediction accuracies. Consider a bigram model that updates conditional probabilities after processing every word and consider what will happen when the model processes an S_3_. Whenever the model processes a T_42_, *P*(2|4) increases and *P*(1|4) decreases. Thus, when the model experiences a string of several T_42_'s, as it does in an S_3_, the accuracy should steadily increase across these and then should plummet on the final T_41_. The observed pattern is very different from this: the first T_42_ and the T_41_ in the first S_3_ have high average accuracies (near 75%) while the second T_42_ average accuracy is 19% (see Supplementary Figure 1). We tested whether the prediction accuracies were different across those three transitions of the first S_3_, using a logit mixed-effects model with TransitionType (first T_42_, second T_42_ [reference level], and T_41_) as a fixed effect as well as a by-subject random interecept. The test suggests that participants predicted the second T_42_ significantly worse than the first T_42_ (*b* = 1.836, *SE* = 0.370, *z* = 4.97, *p* < 0.0001) and T_41_ (*b* = 2.142, *SE* = 0.379, *z* = 5.66, *p* < 0.0001).

In sum, participants learned a novel, deep embedding (S_3_) better when they were trained with sentences in a recursive relationship (Sequence 1) than when trained with sentences not in a recursive relationship (Sequence 2). This is consistent with our hypothesis of a representational continuum: we hypothesize that those participants trained on Sequence 1 were closer (on the continuum) to the a recursive encoding than those trained on Sequence 2, without actually being at the recursive encoding. Therefore, it took more steps in the test phase for the Sequence 2 participants than the Sequence 1 participants to become capable of handling the deeper level of embedding. However, there is no evidence of spontaneous generalization in this experiment. Given the apparently stable patterns at the second half of the training phase (see Figure 2), the failure of spontaneous generalization is probably not due to short training. Rather, we hypothesized that if Sequence 1 participants had received more information indicating the form of the recursive pattern, they would have spontaneously generalized it.

## 4. Experiment 2

In Experiment 2, we investigated whether participants could spontaneously generalize to one deeper level of embedding if they were exposed to more sentence types holding a recursive relationship. For this purpose, we used a finite language L_3_, with one additional level of embedding (see Table 1). We created a sequence of sentences such that three sentence types S_1_, S_2_, and S_3_ occurred in the training phase and the test phase began with an instance of S_4_. We expected that with exposure to more lower levels of embedding, we would observe spontaneous generalization to higher levels.

### 4.1. Material and methods

#### 4.1.1. Participants

Sixty-nine college students of the University of Connecticut participated in the experiment for course credit.

#### 4.1.2. Materials and design

A sequence of 456 words (SeqType = Sequence 3) was created by concatenating many instances of the four sentence types (S_1_, S_2_, S_3_, and S_4_) as in Experiment 1 where S_4_ = 1 S_3_ 2 3 4 (see Section 1 in Supplementary Materials). The first 304 words (corresponding to the study phase) consisted of S_1_s, S_2_s, and S_3_s^13^.

The last 152 words (corresponding to the test phase) began with an S_4_ and included a mixture of S_1_s, S_2_s, S_3_s, and S_4_s. The sentences with the deepest level of embedding (S_3_ in Sequence 1 [Exp1] and S_4_ in Sequence 3) were not presented during the study phase. Thus, examining the test phase provides an opportunity to examine generalization from lower levels of embedding to higher levels.

#### 4.1.3. Procedure

The procedure was the same as in Experiment 1. Each session took about 20 min.

### 4.2. Results

One participant was excluded from further analyses because the participant's overall mean trial prediction accuracy was very low (*M* = 0.119).

The bottom panel of Figure 2 shows trajectories of the average sentence prediction accuracy (SentAcc) separated by sentence types (SentType) in Experiment 2 (Sequence 3). The average sentence prediction accuracy trajectory of S_4_ in the test phase of the Sequence 3 condition (down-pointing triangles on the bottom panel) look very different from the trajectory of S_3_ in the test phase of the Sequence 1 condition (top-pointing triangles on the top panel), suggesting that a high proportion of participants correctly processed all deterministic transitions of the first instance of novel sentence type S_4_.

Twenty-eight (41%) of 68 participants trained with Sequence 3 correctly predicted the first instance of S_4_. The probability of correctly predicting the first three T_42_s and the last T_41_ of an S_4_ by chance is 0.0625 (= (1/2)^4^). The exact binomial test suggests that 41% successful generalization is above chance, *M* = 0.412, 95% CI = [0.294, 0.538], *p* < 0.0001^14^. Thus, in Experiment 2, unlike in Experiment 1, there is strong evidence of recursive generalization at the first encounter of a new level of embedding.

As in Experiment 1, a close investigation of trial-level prediction accuracy (see Supplementary Figure 2) suggests that when the learners failed to predict a sentence, it was mainly because they failed to predict the penultimate pop transitions (i.e., the last T_42_ [4-to-2 transition] in a sentence). Although the pattern is not expected from a bigram or trigram model, it is expected if the learners have acquired a higher-order *n*-gram model during the training phase.

We define the *n*-gram prediction accuracy for the *t*-th word (*ACC*_*n*_(*t*)) as follows:

$$ACC_{n}\left(t\right)=P\left(W_{t}|W_{t-n+1}\cdots W_{t-1}\right)=\frac{N\left(W_{t-n+1}\cdots W_{t-1}W_{t}\right)}{N\left(W_{t-n+1}\cdots W_{t-1}\right)}$$

where *W*_*t*_ is the *t*-th word in the experiment sequence; and *N*(·) is the count of an *n*-gram observed in the subsequence *W*_1_⋯*W*_*t*−1_ of an experimental sequence. In other words, this model can remember sequences *n* symbols long. At each word in context, it predicts the next word by examining all the preceding circumstance in which there were *n*-1 preceding words matching the current context. It chooses the next symbol by drawing randomly from the four possible symbols in proportion to how often they each occurred in all such preceding length *n*-1 contexts. When the model encounters *n*-1 words that it has never seen before, it reduces the context length until it finds at least one preceding instance. Note that after a single *S*_1_ has occurred, the model can always find a matching *n*-gram for some *n* > 0 because, at that point, all four symbols have occurred.

Although this simple *n*-gram model can make context-dependent predictions, it can never make correct predictions on the first instance of a novel sentence type with a deeper level of embedding. For example, consider a 14-gram model which can make correct predictions from the second instance of S_4_ because at this point, it has already experienced the 13-gram context 1 1 1 1 2 3 4 2 3 4 2 3 4 which was followed by 2. This model has no knowledge of the 13-gram context when it encounters the first instance of S_4_. The model therefore goes down to the 12-gram context 1 1 1 2 3 4 2 3 4 2 3 4 (= S_3_) which it has experienced in the training phase and predicts 1 based on this experience. However, the correct prediction is 2. Thus, the 14-gram model makes a wrong prediction at the penultimate 4 of the first instance of S_4_. This failure to generalize to longer sequences is a general property of *n*-gram models.

The results of Sequence 3 training thus provide evidence of recursive generalization and are not plausibly handled by a finite-state account.

## 5. Gradual recursive generalization

We have so far taken advantage of the fact that the Locus Prediction task gives us detailed information about individual sentences, thus gleaning evidence for first-try generalization. Another advantage of the task is that it allows us to investigate the learning process in detail.

In this section, we focus on the Sequence 3 training, showing (i) that participants progress through a series of stages corresponding to successively more complex finite state approximations of the recursive language in a fashion similar to what Tabor (2003) observed with Fractal Learning Neural Networks. This result is not surprising, for we presented the materials in a correspondingly staged fashion, but it serves as a foundation for further analysis. We then show (ii) that, in making this progression, the participants do not transition discretely from stage to stage—instead, they spend much time in behaviors that seem to be blends of stages. Finally, we providence evidence (iii) that the details of the blends cannot be explained by positing noise added to the finite state models or by assuming probabilistic mixture of these models—instead, the pattern suggests an attractor in the dynamical systems sense. The section thus provides evidence that the representational continuum we have been observing is of the sort commonly observed in nonlinear dynamical systems.

### 5.1. Preparatory analysis

We used a *Grammar Bearing Point Language Classification Algorithm* (GBPLCA) (see Section 2 in Supplementary Materials) to obtain a sequence of grammars from each individual. The algorithm uses sentence-level response patterns to hypothesize a grammar after each sentence has been processed by each participant. It assumes that an individual's grammar is updated right after the individual has processed a sentence and the updated grammar is reflected in the individual's response to the next sentence input. The basic idea is to compare empirical profiles of trial-level prediction accuracy on different sentence types with model profiles (see Table 2) and, for each sentence that each participant processed, to assign the participant to a model that fits the participant's behavior up to that point if possible^15^. A model profile, FiniteM, for a level-N sentence (where M ≤ N) is expected under a finite-state grammar G_*M*_ consisting of M symbolic rules that generate S_1_, ⋯ , S_*M*_. We also consider the recursive grammar G_R_ which generates all the sentences of the target language. We will refer to G_1_ through G_4_ and G_R_ as *bearing point grammars*.

**Table 2:** **Model profiles of trial-level prediction accuracy motivated by symbolic grammars**.

	**S_1_**	**S_2_**	**S_3_**	**S_4_**
	**1 2 3 4**	**1 1 2 3 4 2 3 4**	**1 1 1 2 3 4 2 3 4 2 3 4**	**1 1 1 1 2 3 4 2 3 4 2 3 4 2 3 4**
Finite1	* 1 1 1	** 1 1 0 1 1 1	*** 1 1 0 1 1 0 1 1 1	**** 1 1 0 1 1 0 1 1 0 1 1 1
Finite2		** 1 1 1 1 1 1	*** 1 1 1 1 1 0 1 1 1	**** 1 1 1 1 1 0 1 1 0 1 1 1
Finite3			*** 1 1 1 1 1 1 1 1 1	**** 1 1 1 1 1 1 1 1 0 1 1 1
Finite4				**** 1 1 1 1 1 1 1 1 1 1 1 1

*Note: A 0 indicates that the participant got the prediction wrong. A 1 indicates that the participant got the prediction right. The asterisks under the word-1 columns indicate that the predictions of nondeterministic transitions are ignored*.

We assumed that all symbolic grammars predict 3 after 2 and 4 after 3 because 2 is always followed by 3 and 3 is always followed by 4 in the experimental language. We ignored the predictions of nondeterministic transitions T_11_ or T_12_ because they cannot be predicted with certainty. Thus, the models of learners can make wrong predictions only when they encounter word 4. After they made wrong predictions on a certain T_42_ in the middle of level-2, 3, 4 sentences, they are assumed to predict 1 after 4. This behavior guarantees that all models make correct predictions of the T_41_ occurring at the end of all sentence types. With these assumptions, learners with symbolic grammars can be different only in terms of their prediction accuracy on T_42_s.

The algorithm takes a sequence of sentence-by-sentence prediction accuracy profiles (e.g., [0 0 1 1][ 0 0 1 1 1 1 1 0] …) from each individual and returns a sequence of grammars (e.g., G_1_, G_0_, G_0_, G_1_, …, G_1_, G_2_, …) that we refer to as an individual *grammar trajectory*. Specifically, for each new sentence, the algorithm checks to see if it matches a profile in Table 2. In the case of a match, and if the performance on the sentence is perfect, then the person at that time is assigned either the status corresponding to the perfect match (status G_*k*_ corresponds to row *k* of the table) or the preceding status of the participant, whichever is higher. In the case of a match on row *k* where the performance was not perfect, the algorithm checks the most recent instances of all sentences of other lengths. If the person has done at least as well as G_*k*_ on each other sentence length, then the status G_*k*_ is assigned. In all other cases, the status G_0_ is assigned. A special case applies to *G*_*R*_: in addition to the above criteria, the only way that a participant can be assigned status G_R_ is to perform perfectly the first time he or she encounters a sentence at level *k* for *k*≥1. In sum, the algorithm assigns status G_*k*_ if a person has most recently demonstrated G_*k*_ competence, but any aberrant behavior results in assignment to G_0_.

Because the data can only be collected at discrete time points, the data gathered will inevitably give the impression of a series of saltations. It is possible for the data to look this way when, in fact, the mental trajectory that gives rise to them is continuous. Although we cannot explicitly demonstrate continuity, we can demonstrate intermediacy, and intermediacy is a kind of indirect evidence for continuity. Specifically, we will show that learners progress through a series of stages, G_1_, G_2_, G_3_, G_4_, G_R_, each of which is described by a simple grammar and the grammars are naturally ordered by the length of the dependencies they track. Moreover, we will present evidence that between the first four stages, we can observe behaviors that are manifestly intermediate between them, though not in a simple averaging sense, thus revealing a non-trivial continuity underlying the discrete differences.

**Figure 3:**
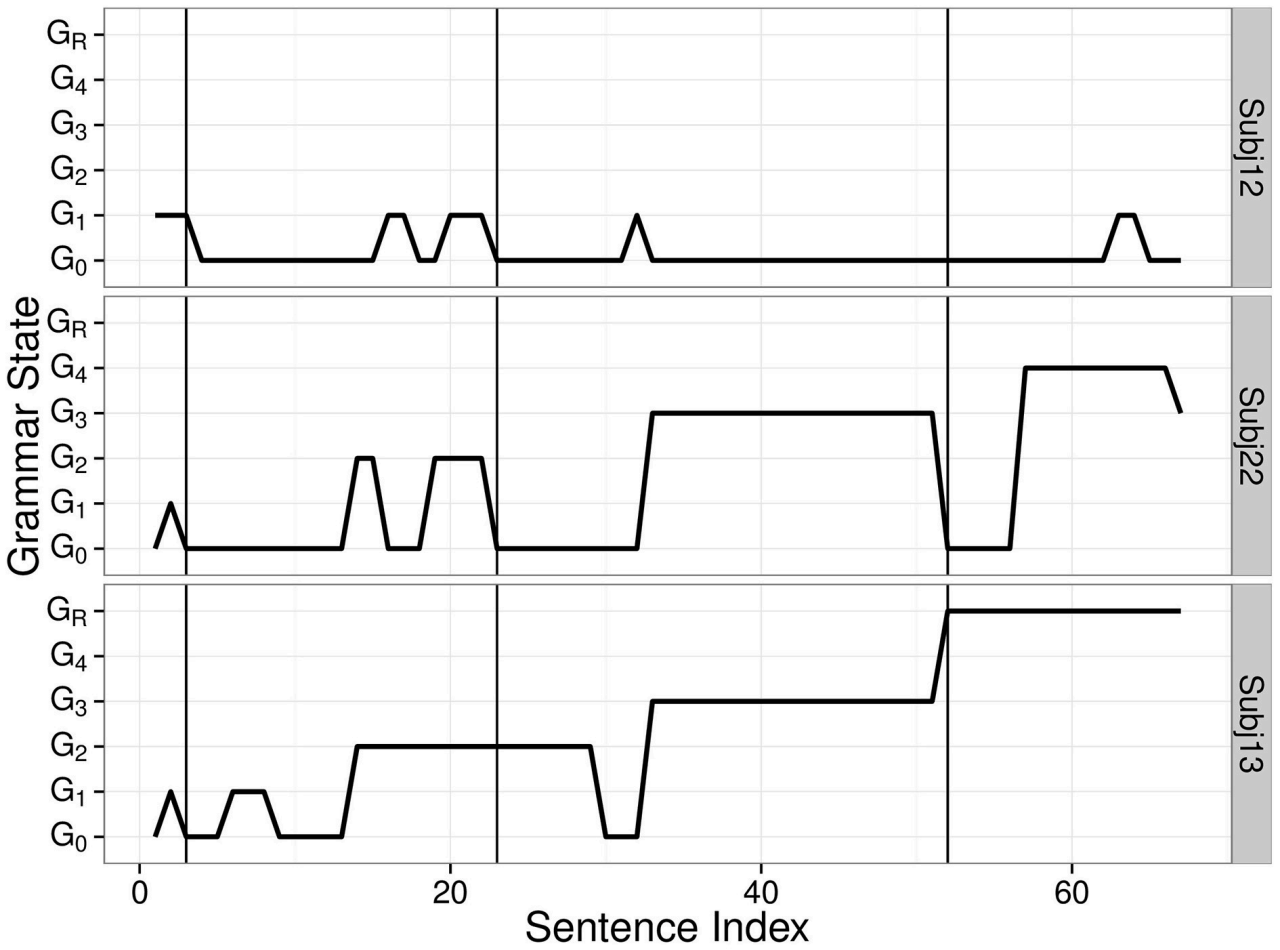
**Sample trajectories of individual grammar changes**. Three vertical lines indicate where the first instances of S_2_, S_3_, and S_4_ were introduced.

Figure 3 presents sample trajectories of individual grammar changes. The top panel suggests that Subj12 predicted the next word mainly based on a simple rule S → 1 2 3 4. The middle panel suggests that Subj22 learned the sentence structure of all four sentence types but did not spontaneously generalize to a deeper level of embedding. The bottom panel presents the grammar change of an individual who learned recursion. The transition from G_3_ to G_R_ (observed around the third vertical line) tells us that the individual correctly processed the first instance of a novel S_4_.

### 5.2. Result (i): Stages of learning

Now we check to see if learners progress from grammars with lower levels of embedding to grammars with higher levels of embedding, in line with the Fractal Learning model of Tabor (2003). For each participant, a transition count matrix was constructed with the G_0_s ignored. For example, the series of transitions G_1_ → G_0_ → G_2_ was treated as the transition G_1_ → G_2_. The progression hypothesis predicts that the number of transitions in the upper triangle (G_*j*_ → G_*k*_ where *j* < *k*) be greater than the number of transitions in the lower triangle (G_*k*_ → G_*j*_ where *j* < *k*) of the transition count matrix, ignoring the diagonal elements. A paired-samples *t*-test revealed that the number of transitions from grammars with less embedding to grammars with more embedding (*M* = 2.52, *SD* = 1.32) was significantly greater than the number of transitions in the opposite direction (*M* = 0.84, *SD* = 0.90), *M*_*diff*_ = 1.69, *t*_(66)_ = 13.00, *p* < 0.0001, supporting the progression hypothesis^16^.

### 5.3. Result (ii): Intermediate behaviors

In this section, we explore the G_0_ states, noting that they often occur between the Bearing Point grammars and often mediate the transition from one Bearing Point grammar to another. For each participant, we constructed a transition count matrix again, this time including G_0_s. The matrix of transition counts aggregated across all participants is presented below:

$$\begin{array}{c} \sum_{i}c_{i}=\begin{array}{c} \;\begin{array}{cc} \begin{array}{cc} \begin{array}{cc} \begin{array}{cc} \begin{array}{cc} \;G_{0} & \;G_{1} \end{array} & \;G_{2} \end{array} & G_{3} \end{array} & G_{4} \end{array} & \;G_{R} \end{array}\\ \begin{array}{c} G_{0}\\ G_{1}\\ G_{2}\\ \begin{array}{c} \begin{array}{c} G_{3}\\ G_{4} \end{array}\\ G_{R} \end{array} \end{array}\left(\begin{array}{cccc} \begin{array}{c} \begin{array}{c} 2023\\ 163 \end{array}\\ 65 \end{array} & \begin{array}{c} \begin{array}{c} 150\\ 504 \end{array}\\ 16 \end{array} & \begin{array}{c} \begin{array}{c} 60\\ 40 \end{array}\\ 486 \end{array} & \begin{array}{c} \begin{array}{c} \begin{array}{cc} \begin{array}{cc} 52 & 24 \end{array} & 10 \end{array}\\ \begin{array}{cc} 4 & \begin{array}{cc} \;2 & \;1 \end{array} \end{array} \end{array}\\ \begin{array}{cc} 15 & \begin{array}{cc} 2 & \;5 \end{array} \end{array} \end{array}\\ 40 & 6 & 3 & \begin{array}{cc} 397 & \begin{array}{cc} 7 & 16 \end{array} \end{array}\\ 15 & 1 & 1 & \begin{array}{cc} 3 & \begin{array}{cc} 146 & \;0 \end{array} \end{array}\\ 19 & 1 & 2 & \begin{array}{cc} 1 & \begin{array}{cc} 0 & 208 \end{array} \end{array} \end{array}\right) \end{array} \end{array}$$

where the cell (G_j_, G_k_) indicates transitions from G_j_ to G_k_ For example, the aggregated number of transitions from G_1_ to G_0_ was 163.

The matrix reveals a large number of cases in Row 1 and Column 1 [even with cell (1, 1) not counted]. In fact, about 83 percent of the off diagonal elements lie in Row 1 or Column 1. This suggests that the transitions between states G_1_, G_2_, G_3_, G_4_, and G_R_ happen largely via the G_0_s. In other words, the G_0_s are transitional between the other Bearing Point Grammar states. We call this the “Intermediate States Hypothesis.” The Intermediate States Hypothesis claims that the bearing point grammars lie in a connected space of possible distributional patterns and that the learner travels fairly directly (i.e., approximately linear interpolation) between the distributions when transiting from one bearing point grammar to the next one. However, there is a confound. It could be that the main learning behavior is a stepwise progression from G_1_ to G_R_ but the behavior is very noisy, and the G_0_s arise from the noise. In particular, one might assume that the learner's grammar (G_1_, G_2_, G_3_, G_4_, or G_R_) makes a prediction, but the learner chooses between this prediction and the uniform distribution across all four symbols with some probability. If the G_0_s are just noisy versions of the Bearing Point models in this sense, then they should show deviance from high accuracy relative to those models on *all* transitions. We call this the “Progression + Noise Hypothesis.” In the following, we analyze the distribution of G_0_ states, finding it consistent with the Intermediate States Hypothesis and inconsistent with Progression + Noise.

#### 5.3.1. Interpolation analysis: Graphical

First, we investigated how participants responded to S_4_ when their grammatical knowledge was classified into two different cases of G_0_s, ones observed between the first occurrence of G_2_ and the first occurrence of G_3_ and ones observed between the first occurrence of G_3_ and the first occurrence of G_4_ or G_R_ in individual grammar trajectories. We refer to the two groups as G_0_(G_2_, G_3_) and G_0_(G_3_, G_4_/G_R_), respectively. There was no case of G_0_ included in both G_0_(G_2_, G_3_) and G_0_(G_3_, G_4_/G_R_).

**Figure 4:**
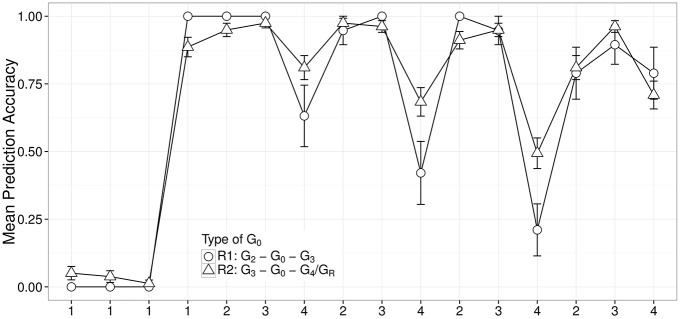
**Plot of mean prediction accuracy across different words of S_4_ separated by two types of G_0_**. G_0_(G_2_, G_3_) corresponds to a set of G_0_ observed between the first G_2_ and the first G_3_ in each individual; G_0_(G_3_, G_4_/G_R_) corresponds to a set of G_0_ observed between the first G_3_ and the first G_4_ or G_R_ in each individual.

We focused on S_4_ because this sentence type was presented only in the test phase when participants were least likely to guess next words randomly. We considered grammars G_0_(G_2_, G_3_) and G_0_(G_3_, G_4_/G_R_) because they could not correctly process S_4_ so we expect more chances to observe systematic errors. The first set contained 12 response vectors and the second set contained 27 response vectors. Average response patterns to S_4_s when learners were at G_0_(G_2_, G_3_) and G_0_(G_3_, G_4_/G_R_) are presented in a parallel coordinate plot (see Figure 4).

The fact that the deviations in Figure 4 are concentrated on the 4-transitions, which are the points where the grammar needs to change at each stage in order to move to the next stage, supports the Intermediate Stages hypothesis.

#### 5.3.2. Interpolation analysis: Statistical

Next we examined the structure of the distribution of grammar states in the test phase, assuming that individual learners' grammars did not change much in the test phase.

The Progression + Noise Hypothesis predicts that G_0_s should be random distortions of Bearing Point Grammars so individual learners should show the same amount of variation in prediction accuracy across all transitions^17^. By contrast, because it claims that the transitional states are approximately linear mixtures of the bearing point grammar distributions, the Intermediate States Hypothesis predicts that individual learners should show greater variation on transitions (e.g., T_42_s) in which the Bearing Point Grammars (G_1_ to G_R_) contrast than on transitions in which all of them agree (e.g., T_23_ and T_34_s)^18^. To test this statistically, we applied the variance vector analysis of Cho et al. (2011) to the test phase data.

First, we created an individual variance vector for each individual, **V**_*T*_(*i*) = ($v_{i}^{\left(1\right)}$, $v_{i}^{\left(2\right)}$, ⋯ , $v_{i}^{\left(j\right)}$, ⋯ , $v_{i}^{\left(40\right)}$), where $v_{i}^{\left(j\right)}$ is an individual *i*'s variance of trial-level binary prediction accuracy across different instances for the *j*-th transition type in the test phase; forty transition types (4 from level-1, 8 from level-2, 12 from level-3, and 16 from level-4) occurred in the test phase.

Second, we created a global variance vector as follows. (a) A model vector of binary prediction accuracy across 40 transition types was defined for each of four Bearing Point grammars based on Table 2 as well as an additional Bearing Point grammar, G_RND_^19^. G_RND_ corresponds to a grammar state at which an individual randomly chooses one of four boxes so the expected trial-level accuracy at G_RND_ is 0.25 for all transition types. We assumed that learners were at G_RND_ at the beginning of the experiment. (b) A vector, **M**_*T*_ = (*m*^(1)^, ⋯ , *m*^(40)^), was created such that *m*^(*j*)^ is the expected prediction accuracy for the *j*-th transition type averaged across the five model vectors. (c) Assuming a Bernoulli distribution, a global variance vector, **V**_*T*_ = (*v*^(1)^, ⋯ , *v*^(40)^), was created such that *v*^(*j*)^ = *m*^(*j*)^(1 − *m*^(*j*)^).

Our hypothesis predicts that **V**_T_(*i*) would be more aligned with **V**_T_ than with a uniform variance vector, **V**_U_ = (1, 1, ⋯ , 1). The cosine of the angle between **V**_T_(*i*) and **V**_T_ would be greater than the cosine of the angle between **V**_T_(*i*) and **V**_U_. A paired-samples *t*-test revealed that the former (*M* = 0.432, *SD* = 0.216) was significantly greater than the latter (*M* = 0.397, *SD* = 0.236), *M*_*diff*_ = 0.036, *t*_(67)_ = 3.95, *p* < 0.001, showing that individual variability is not uniformly distributed around bearing point grammars but aligns with the dimensions on which the bearing point grammars differ. This result is consistent with our hypothesis that there is a connected manifold that links the bearing point grammars to one another^20^.

### 5.4. Result (iii): Evidence for dynamical stability

The intermediate states show a subtle property that allows us to obtain some further information about the nature of the system. Consider another kind of noise model that is capable of describing intermediate stages: when the system is in transition between G_*k*_ and G_*k*+1_ (*k* = 1, 2, 3) or between G_3_ and G_R_, it probabilistically chooses between the two grammars at each word-transition. This model, which we call “Probabilistic Mixture of Bearing Points” predicts profiles in which the noisy behavior is limited to the points of differentiation between the two grammars. Indeed, such an account is roughly consistent with the profiles shown in Figure 4, thus improving on the Progression + Noise account. However, we will present evidence that Probabilistic Mixture fails to capture a distinctive pattern in the data. In Figure 4, the mean prediction accuracy on the last T_23_ seems lower than the mean prediction accuracy on the last T_34_ although both transitions are equally easy to predict. It suggests that there are interactions between nearby trials in the sequence such that the experience of the participant on one trial can influence what happens on successive trials in ways that are not captured by the grammar predictions about those trials. We hypothesized that an error has a perturbing effect: it should knock the system off the path of correct prediction on the trials immediately following the error (T_23_s and T_34_ trials), but the degree of distortion should diminish as the trials progress. We call this account “Dynamical Stability.”

To test the hypothesis, we first compared trial-level prediction accuracy for T_23_ and T_34_; the transition probability in Sequence 3 is 1 for both transitions. If noise is purely random, prediction accuracy should be roughly equal for both transition types. Binary trial-level prediction accuracy was modeled as a function of WordType (dummy-coded with word 2 as reference level); a by-subject random intercept, a by-subject random slope of WordType, and the correlation between them were included as random effects. The effect of WordType was significant, *b* = 0.535, *SE* = 0.098, *z* = 5.45, *p* < 0.0001; consistent with the predictions of Dynamical Stability, the odds of correct prediction was 1.7 (= *e*^0.535^) times greater for T_34_ than for T_23_.

Note that T_23_s follow more difficult-to-predict transitions T_42_s (requiring processing nonadjacent dependency) and T_12_s (involving uncertainty) while T_34_s follow very easy-to-predict T_23_s^21^.

This result is consistent with Dynamical Stability, but it does not take into account the hypothesized perturbing events: the errors on T_42_s. To test the claim that these errors were the primary source of errors on following trials, we took prediction accuracy on T_23_s right after T_42_s occurring in S_2_s, S_3_s, and S_4_s in the experimental sequence and classified the data into two groups depending on prediction accuracy on the previous T_42_ (PrevAcc = 0 vs. 1). We used a logit mixed model to test if prediction accuracy on T_23_ after T_42_ was a function of PrevAcc; a by-subject random intercept, a by-subject random slope of PrevAcc, and their interaction were included as random factors. The effect of PrevAcc was significant, *b* = 3.444, *SE* = 0.464, *z* = 7.43, *p* < 0.0001; the odds of correctly predicting T_23_ was 31.31 (= *e*^3.444^) times greater after correct predictions of T_42_ than after wrong predictions of T_42_, supporting the idea that a prediction error tends to perturb a learning system such that it makes a prediction error on the next transition although the transition is very easy to predict. Similarly, the odds of correctly predicting T_34_ was 34.17 (= *e*^3.531^) times greater right after correct predictions of T_23_ than right after wrong predictions of T_23_, *b* = 3.531, *SE* = 0.270, *z* = 13.10, *p* < 0.0001^22^.

It is clear that the mixture model cannot explain these Dynamical Stability results because all bearing point grammars considered in the mixture model predict T_23_ and T_34_ with certainty so any non-noisy mixture of the grammars must predict those transitions correctly and in a noisy mixture, the expected rate of failure should be equal for both transition types.

## 6. General discussion

In Experiment 1, we presented evidence that human learners are sensitive to recursive structure underlying a sequence of box locations. The participants trained on low embedding levels could learn a higher level more quickly than the participants trained on a non-recursive pattern. In Experiment 2, we provided evidence of spontaneous recursive generalization. When participants were exposed to level-1, 2, 3 sentences in the training phase, about 40% of participants correctly processed the first instance of level-4 sentences newly introduced in the test phase. Experiments 1 and 2 together suggest that human learners can achieve recursive generalization in the Locus Prediction task.

There is some evidence that human learners and probably some nonhuman animal learners can learn at least counting recursion (Fitch and Hauser, 2004; Perruchet and Rey, 2005; Bahlmann et al., 2006; Friederici et al., 2006; Gentner et al., 2006; De Vries et al., 2008) and human learners can learn even mirror recursion (Bahlmann et al., 2008; Lai and Poletiek, 2011, 2013). However, we find that these studies did not provide strong evidence of recursive generalization, that is generalization to at least one deeper level. In most of these studies, generalization behavior was tested by investigating the learners' ability to recognize novel instances at the trained levels-of-embedding but that type of generalization does not necessarily require recursive rules. In the studies that tested generalization to deeper levels of embedding (for example, Gentner et al., 2006; Lai and Poletiek, 2011, 2013), we noted that the result might have stemmed from learning that occurred in response to test stimuli. A finite-state model like an *n*-gram model can learn quickly after the first instance of a novel sentence type. The present study provides much stronger evidence of recursive generalization.

In the Gradual Recursive Generalization section, we provided evidence that individual grammar systems metamorphose gradually by investigating the training phase data as well as the test phase data from Experiment 2—as far as we know, our study is the first detailed investigation of grammar change in human recursion learning. More specifically, we showed three aspects of developmental changes in recursion learning. First, individual learners' grammars progressed from a grammar with low embedding levels to a grammar with high embedding levels toward the ideal recursive grammar. Second, the interpolation analysis suggests that grammar systems lie on a continuum and individual learners explore this continuous grammar space to acquire a grammar that can handle the language. In other words, individual grammars do not “jump” from finite-state grammars to the recursive grammar; rather, they change gradually from finite-state grammars to the recursive grammar through intermediate grammars. Third, participants made more prediction errors even on the very-easy-to-predict transitions (e.g., T_23_ and T_34_) right after they had made prediction errors, supporting the dynamical stability account: A language processing system recovers gradually from the perturbation induced by an unexpected word while processing familiar events. A mixture model of bearing point grammars cannot explain this result because all bearing point grammars correctly predict those transitions.

In sum, our study suggests that (a) participants are sensitive to recursive structure in a language (Experiment 1); (b) they can learn counting recursion such that they could generalize beyond experience (Experiment 2) if they were exposed to level-1 through level-3 sentences; (c) they learn recursion gradually from a bearing point grammar with lower embedding levels to another bearing point grammar with higher embedding levels through some intermediate grammars (Gradual Recursive Generalization); and (d) they recover from their prediction errors gradually (Dynamical Stability).

What we observed on adults' artificial language learning, gradual grammar change, is consistent with neural network studies with English-like artificial languages. Elman (1991, 1993) observes the Simple Recurrent Network can learn an English-like artificial language including center-embedding sentences if the model is trained with the “staged input” (simpler sentences earlier and complex sentences later) or if the model's memory is limited early in the training. It implies that the system develops the knowledge of simpler structures earlier than the knowledge of more complex structures. Although the effect of “starting small” is under debate (Rohde and Plaut, 1999), a gradual development of knowledge is a general property of dynamical systems models and, if such models can explain human language acquisition, of language acquisition. In our study, we reported behavioral evidence for intermediacy in knowledge development, contributing to the literature of rule learning and possibly of language acquisition. We note that our findings do not weight in for or against the Starting Small hypothesis because we did not contrast conditions with and without Starting Small.

We focus now on possible explanations for the intermediacy observed in the section on Gradual Recursive Generalization. We noted in the Introduction that in a situation such as the present one, where we have evidence that participants are progressing through a series of structured forms which correspond to simple-to-specify grammatical systems, it is natural to hypothesize a probabilistic grammar blending account. This view corresponds to the Probabilistic Mixture of Bearing Points (PMBP) hypothesis which we considered in the section “Result (iii): Evidence for Dynamical Stability.” Such an approach is natural to consider in light of the effectiveness of the simple computational bearing points in describing the progression discussed in the section “Result (i): Stages of Learning” when faced with the evidence portrayed in Figure 4 of intermediate behavior. Such an approach is also consistent with the currently widely adopted strategy in cognitive modeling of identifying a set of candidate computational mechanisms and then using Bayesian inference to discover probabilistic mixtures of mechanisms which make the data probable in light of the evidence (Perfors et al., 2011). Nevertheless, we argued against probabilistic mixture in the section “Result (iii): Evidence for Dynamical Stability,” noting that the intermediate grammar states (the G_0_s) include perturbation-related distortion of behavior on 2–3 and 3–4 transitions, which is not predicted by PMBP.

Now we consider the question more fully. We do not think it is possible to entirely reject PMBP based on the evidence present so far. It is true that PMBP does not predict the Dynamical Stability effects on its own. Nevertheless, one might hypothesize that there is some independent feature of the processing that results in the perturbation effects: perhaps the perturbations are like garden path situations in sentence processing—people expect 1 after 4 but they get 2 and then they have to reanalyze, which takes time. Before the reanalysis completed, they are stuck with their previous interpretation so they make errors on succeeding words until the reanalysis is done. If the time it takes for reanalysis varies across participants (following, for example, an exponential distribution), then the pattern of decreasing errors after perturbation that we observed would be expected. We think this model should be carefully considered since it involves minimal divergence from well established ideas in the field. At the same time, we take this opportunity to articulate a different approach, inspired by the perturbation data, which may also be worth carefully considering. The alternative is motivated by a concern about teleology: the PMBP model predicts the transitional behaviors by taking a weighted average of a less complex bearing point grammar (e.g., G_2_) and a more complex bearing point grammar (e.g., G_3_), and gradually changing the weighting as learning progresses. In order to perform this calculation, it must, in the implementation we suggest, identify the more complex bearing point grammar as soon as it begins to slightly diverge from the less complex bearing point grammar. This amounts to a kind of teleology in the sense the endpoint is recognized long before it is reached. In the case of children learning natural language, it is plausible that long evolutionary sculpting might have established in the brains of infants a Universal Grammar containing many bearing point grammars with respect to which learners could interpolate (see Perfors et al., 2011). In the case of our artificial language, it is less plausible to claim that people are born with the bearing points of this particular domain. One interesting feature of dynamical models like the Simple Recurrent Network (Elman, 1990) and the Fractal Learning Neural Network (Tabor, 2003, 2011) is that they characterize the grammatical structure itself as a point in a connected space (Tabor et al., 2013b), rather than characterizing the probabilistic weighting of grammars as a point in a connected space, as PMBP does. This has the consequence that the model can be expressed in simple, “sub-symbolic” terms—for example, as weights on connections between nodes in a network, rather than in terms of an inventory of possible structures. Now, it is true that, in principle, such a characterization might be behaviorally equivalent to a mixture-of-structures model—it might predict exactly the same range of behaviors with same probabilities. However, there is a constraint in the mixture of structures approach which does not necessarily hold in the dynamical approach: probabilistic mixture is a form of linear mixture. Dynamical neural networks generally perform nonlinear mixture—that is, they interpolate between observed behaviors with nonlinear manifolds (Tabor, 1995). This means that particular dynamical models make different predictions about trajectories of learning than probabilistic mixture models. Tabor (1995) presents evidence that such nonlinear mixture is more suited to characterizing certain historical change episodes than linear mixture approaches. The same approach to empirical distinction may allow us to tease apart models of artificial language learning. We leave this as a topic for future research.

We conclude that recursive systems lie on a continuum of grammar systems which are organized so that grammars that produce similar behaviors are near one another. People learning a recursive system are navigating progressively through the space of these grammars exhibiting evidence that they may embody a dynamical system with a special kind of grammar-dependent attractive set. The dynamical framework offers an interesting nonlinear alternative to probabilistic mixture models and may be worthy of further investigation.

## Author contributions

ES and WT designed the experiments. ES performed the experiments. PC analyzed the data and wrote the manuscript. WT contributed to data analyses and to manuscript writing. All authors reviewed the manuscript.

### Conflict of interest statement

The authors declare that the research was conducted in the absence of any commercial or financial relationships that could be construed as a potential conflict of interest.

## Footnotes

^1^Fitch and Friederici (2012) rightly note that *a*^*n*^*b*^*n*^ need not be generated with a rule that matches the first A with the last B, the second A with the second-to-last B, etc.—other schemes are possible. They argue that, because of this, *a*^*n*^*b*^*n*^ is not definitively a recursive language. We do not think much hangs on the question of this terminology—what matters is the fact that a finitely defined processor of the language needs to employ a self-referential memory principle. We include *a*^*n*^*b*^*n*^ in the class of “recursive” languages because the weakest Chomsky Hierarchy class capable of generating it has to employ a rule that calls itself, either directly or indirectly. We do not claim that the successful learners of the language learned the recursive rules. As pointed out by Fitch and Friederici (2012), participants may have learned a count-and-match strategy, although we think the count-and-match strategy as well as counting itself requires recursive generalization.

^2^See Tabor (2015) for an extension of the definition of “attractor" to this case.

^3^Sometimes, the term *stage* implies discontinuity or abrupt transition between ordered states (Van der Maas and Molenaar, 1992). In this study, we use the term to distinguish among different periods in learning in which an individual's behavior seems to be generated by different symbolic grammars which are ordered such that each successive grammar handles longer dependencies than the previous one. By the use of the term “stage,” we do not imply discontinuity from one stage to the next.

^4^In this paper, we use a level *k* to refer to the number of *a*s (or *b*s). Note that a level-1 sentence has a sentence with 0 level of embedding.

^5^The task is same as the prediction task used in Cleeremans and McClelland (1991) but we distinguish ours from other prediction tasks used in the SRT paradigm for the following reasons. In our study, there is no separation between training/learning and test/evaluation phases. In prior SRT studies, researchers have used the prediction task for evaluation purposes only and tried to prevent participants from developing knowledge while they are performing the prediction task. We use the prediction task to drive the development of grammatical knowledge and we can infer many details of the forms of this knowledge by observing participant's predictions at every time step.

^6^This scheme has the property of encouraging participants to predict all deterministic transitions correctly and to predict 2 after 1, even though either 1 or 2 can occur after 1 at non-deterministic transitions. Nevertheless, participants are not penalized for failing to predict 2 after 1. In future work, it may be helpful to explore the variant in which participants are penalized every time they fail to predict 2 after 1.

^7^We used one sequence for each condition to reduce variance in our performance measure; we do not think that changing the order of the sentences while preserving the structure thus described would have a noticeable effect on the outcome. Also lower embedding levels were always introduced earlier than higher embedding levels and there were more lower embedding levels than higher embedding levels in the beginning of the sequence. This was intended to make learning easier (see Lai and Poletiek, 2011, 2013). In a subsequent study (Tabor et al., 2014), we observed similar learning when we presented randomly different versions of a stationary model sequence, suggesting that the result reported below is not due to the specific sequence that we used.

^8^In this paper, we focus on accuracy analyses. Data gathered in the locus prediction paradigm contains additional useful information: one can also examine which box participants pick when they deviate from accurate prediction (Tabor et al., 2012b) and reaction times.

^9^The chance level of making all correct predictions on an instance of S_3_ is much lower in the Sequence 2 condition than in the Sequence 1 condition. Note that in Sequence 2, 2 can be followed by 3 or 4; 3 can be followed by 1, 2, or 4; 4 can be followed by 1, 2, or 3. Thus, participants have a 50, 33, or 33% chance of making a correct prediction given word 2, 3, or 4, respectively. Because each of words 2, 3, 4 occurs three times in an S_3_, chance level is equal to ((1/2)(1/3)(1/3))^3^ (= 0.00017). An exact binomial test suggests that 0% of success was not significantly different from chance.

^10^All models investigated in this study were fit using the lmer function included in the R package lme4(Bates et al., 2015) with the maximum likelihood estimation method.

^11^A model with the interaction term between IndexS3 and SeqType was not selected because a likelihood ratio test revealed that adding the interaction between IndexS3 and SeqType to the model with SeqType and IndexS3 as fixed effects did not decrease deviance significantly, $\chi_{\left(1\right)}^{2}=1.46$, *p* = 0.227.

^12^When a correlation between the random intercept and the random slope was assumed, the model failed to converge.

^13^The first instance of the highest-level sentence erroneously appeared four words (from an *S*_1_) later in Sequence 3 than in Sequence 1. Given the stability in performance on Sequence 1 at the end of the training period (Figure 2), it is highly unlikely that this difference could explain any difference in generalization behavior between two conditions.

^14^Even considering only the 14 participants among the 68 who got every S_4_ right through the test phase (that is, those whose correct performance on S_4_'s was especially stable), the exact binomial test with the same chance-level proportion of 0.0625 (note that this criterion is very conservative) indicates that the proportion of successful generalization is above chance, *p* < 0.0001.

^15^We do not claim that the participants actually learned such symbolic finite-state or recursive grammars. When we classify an individual learner's grammar into G_k_, we simply mean the learner's response pattern matched the behavior of the bearing point grammar. Similarly, when we classify an individual's grammar into G_R_, we do not mean the learner acquired specific recursive “rules.” Rather, we mean the individual achieved recursive generalization to novel sentence types.

^16^One participant was excluded from the analysis because the participant's grammar trajectory consisted only of G_0_s.

^17^When no random noise is assumed, any Bearing Point Grammar other than G_0_ predicts trial-level prediction accuracy of 0 or 1. When random noise is assumed with noise level *q*, the expected trial-level prediction accuracy is *q* or 1−*q*. The variance in this noisy version is same in either case.

^18^This hypothesis predicts that individual learners lie on a manifold connecting different Bearing Point Grammars. For example, assume that a learner is at an intermediate state between G_2_ and G_3_. Based on Table 2, it is expected that the learner's expected prediction accuracy on the last T_42_ of S_3_ is in between 0 and 1. Thus, the variance in trial-level accuracy on that transition should be greater than the variance on other transitions. Note that the transition is where two groups of learners who developed G_2_ or G_3_ differ from each other and this group difference will be captured by the variance of the trial-level accuracy on that transition when prediction data are aggregated across both groups. Thus, the hypothesis naturally predicts that the larger a between-individuals variance is on a transition, the larger a within-individual variance on that transition.

^19^In this analysis, we assumed that a learner at any Bearing Point grammar state always predicts 2 after 1. Because participants were instructed to predict only green boxes (i.e., deterministic transitions), most of them showed a very strong tendency to predict 2 after 1.

^20^For seven participants, all elements of **V**_T_(*i*) were 0. To be conservative, we replaced these individual variance vectors with **V**_U_.

^21^Prediction accuracy for T_34_s was significantly higher than prediction accuracy for T_23_s both when T_23_s and T_34_s following T_12_s were considered, *b* = 0.621, *SE* = 0.154, *z* = 4.02, *p* < 0.0001, and when T_23_s and T_34_s following T_42_s were considered, *b* = 0.482, *SE* = 0.133, *z* = 3.62, *p* < 0.0005.

^22^We ran the same analysis with the test phase data. The effect of PrevAcc was statistically significant, *b* = 5.753, *SE* = 1.722, *z* = 3.34, *p* < 0.001, and the odds of correctly predicting T_23_ was 315.1 (= *e*^5.753^) times greater right after correct predictions of T_42_ than right after wrong predictions of T_42_. Similarly, the odds of correctly predicting T_34_ was 94.52 (= *e*^4.549^) times greater right after correct predictions of T_23_ than right after wrong predictions of T_23_, *b* = 4.549, *SE* = 0.785, *z* = 5.80, *p* < 0.0001).
