# Supplementary material for: Discovery of a Recursive Principle: An Artificial Grammar Investigation of Human Learning of a Counting Recursion Language
Source: Front Psychol. 2016 Jun 8;7:867. doi: 10.3389/fpsyg.2016.00867 (PMC4897795; doi:10.3389/fpsyg.2016.00867)
Supplement: Supplementary file 1 [file Presentation1.PDF]

frontiers

Supplementary Material: Discovery of a Recursive Principle: An Artificial Grammar Investigation of Human Learning of a Counting Recursion Language

Pyeong Whan Cho*, Emily Szkudlarek and Whitney Tabor
*Correspondence:
Pyeong Whan Cho:
pcho4@jhu.edu

1 EXPERIMENTAL SEQUENCES

To save space, each experimental sequence is presented as a sequence of sentences rather than a sequence of words: S_1 = 1 2 3 4; S_2 = 1 1 2 3 4 2 3 4; ; S_2^* = 1 1 4 3 2 4 2 3; S_3 = 1 1 1 2 3 4 2 3 4 2 3 4; S_4 = 1 1 1 1 2 3 4 2 3 4 2 3 4 2 3 4. The pipe symbol indicates the boundary between the study phase and the test phase but no indication of the boundary was given during the experimental presentation.

Sequence 1 (Experiment 1)

S_1 S_1 S_1 S_1 S_1 S_1 S_1 S_2 S_1 S_1 S_1 S_1 S_2 S_1 S_1 S_1 S_2 S_1 S_1 S_2 S_1 S_1 S_1 S_2 S_2 S_2 S_2 S_1 S_2 S_1 S_2 S_1 S_1 S_2 S_2 S_1 S_1 S_2 S_1 S_1 S_2 S_2 S_1 S_2 S_2 S_2 S_1 S_2 S_1 S_2 S_2 S_2 S_1 | S_3 S_1 S_2 S_1 S_1 S_2 S_3 S_1 S_2 S_3 S_1 S_1 S_1 S_3 (S_1)

Sequence 2 (Experiment 1)

Sequence 2 was the same as Sequence 1 except that S_2 was replaced with $S_2{}^*$.

Sequence 3 (Experiment 2)

S_1 S_1 S_2 S_1 S_2 S_1 S_1 S_1 S_2 S_1 S_1 S_1 S_1 S_2 S_1 S_2 S_1 S_1 S_2 S_1 S_1 S_1 S_3 S_1 S_1 S_2 S_1 S_2 S_1 S_3 S_1 S_2 S_3 S_1 S_1 S_3 S_1 S_1 S_2 S_1 S_3 S_1 S_2 S_1 S_3 S_2 S_1 S_1 S_1 S_2 S_1 | S_4 S_1 S_2 S_3 S_1 S_4 S_1 S_3 S_1 S_2 S_1 S_4 S_1 S_2 S_3 S_4 S_1

2 GRAMMAR BEARING POINT LANGUAGE CLASSIFICATION ALGORITHM

We consider a model profile of trial-level prediction accuracy, `FiniteM`, for a level-N sentence which is well described by a finite-state grammar G_M consisting of M rules that generate $S_1, \cdots, S_M$. An individual with G_M behaves as follows: First, she always predicts 2 after 1 given the instruction and the nature of feedback; although we describe her prediction responding to word 1, the following algorithm ignores the prediction accuracy on those nondeterministic transitions so this arbitrary choice does not influence the algorithm's product. Second, she treats a subsequence of symbols in level-N sentence which matches a level-M sentence ($M < N$) as a level-M sentence. Third, she predicts 1 after 4 occuring after the subsequence. For example, consider an individual who has knowledge of level-1 sentence but does not have knowledge of level-2 sentence. The individual is assumed to treat the subsequence of 1 2 3 4 nested in a Level-2 sentence [1 [1 2 3 4] 2 3 4] as if it was an actual level-1 sentence. Then, the individual would predict 1 after the first 4 because a new sentence is expected after a level-1 sentence. This is a wrong prediction. After a wrong prediction is made, the individual takes the strategy to predict 1 when

encountering the next 4's until she encounters 1 which indicates the beginning of a new sentence. This individual's prediction accuracy profile would be (0,1,1,1,0,1,1,1) and this profile for S_2 would be classified into Finite1. See Table 2 in the main article for other profiles under consideration.

Let `Seq` be a sequence of sentences. `Seq` is initially set to the experimental sequence but it will be updated by removing its first sentence, `Seq(1)`, during running this algorithm. Let `Lv` be the level of `Seq(1)`; `Lv` would be 1 if `Seq(1)` is the sentence without embedding (i.e., S_1). Let `AnsLv` be the level of the response to `Seq(1)`. By the response to `Seq(1)`, we mean a vector of binary prediction accuracy for all *deterministic* transitions of `Seq(1)`. If the response vector is the same as a model profile `FiniteN`, `AnsLv` is set to `N`. If the response vector does not coincide with any FiniteN vector, `AnsLv` is set to 0. For example, the response vector to level-2 sentence might be (1,0,1,1,1,1) from (**1**,**0**,1,0,1,1,1,1) with prediction accuracy of nondeterministic transitions as boldface. No FiniteN vector equals this response vector. In this case, `AnsLv` would be 0. Let `GH(SentNo)` be a grammar underlying an individual response pattern estimated after the individual processed the `SentNo`-th sentence in a sequence; GH corresponds to an individual's grammar trajectory over the course of learning. The following algorithm takes a sequence of trial-level prediction accuracy data and returns a sequence of grammars. The grammar classification algorithm is presented below:

1. Create a vector of length 0 `Resp` in which the n-th element represents `AnsLv` to the most recent level-n sentence so far. Set `SentNo` and `FlagFirst` to 0.
2. Increase `SentNo` by 1. Check if `Seq(1)` is the first instance of level-`Lv` sentence where `Lv` indicates a level of embedding of the sentence. If so, increase the length of `Resp` by 1 and set a variable `FlagFirst` to 1. In other words, the length of `Resp` increases whenever the algorithm encounters a novel sentence type.
3. Check `AnsLv` for the current `Seq(1)` and replace the `Lv`-th element of `Resp` with `AnsLv`. In Steps 2 and 3, it is assumed that the first instance of level-`Lv` sentence occurs after the first instances of the sentence types with lower levels (1, 2, $\cdots$, `Lv`-1) of embedding.
4. If any element of `Resp` is 0, set `GH(SentNo)` to 0 which does not correspond to any symbolic grammar under consideration.
5. If no element of `Resp` is 0, find the maximum `k` such that `Resp(1) = 1, ..., Resp(k) = k`, where `k` should be equal to or less than the length of `Resp`. It suggests that the individual correctly recognized level-`k` and all the lower level sentences. If `k` equals the length of `Resp` (suggesting that the individual correctly predicted all deterministic transitions of the most recent instances of all sentence types) and `FlagFirst` equals 1 (suggesting that the individual correctly processed all deterministic transitions of the first instance of a novel sentence type), set `GH(SentNo)` to 5 which corresponds to the target recursive grammar G_R. In all the other cases, set `GH(SentNo)` to `k` which corresponds to a finite-state grammar G_k.
6. Set `FlagFirst` to 0; update `Seq` by removing `Seq(1)` from it; if Seq is null/empty, then terminate; otherwise, go to 2.

For example, if an individual had processed the sentences $\cdots$ S_2^{19} S_1^{20} S_1^{21} S_1^{22} S_3^{23} where the superscripts indicate the sentence indices in the experimental sequence (and the subscripts indicate the embedding level as before), the individual's grammar after processing S_3^{23} was decided based on the prediction accuracy profile on three sentences S_1^{22}, S_2^{19}, and S_3^{23}; by that sentence, no S_4 had been presented so the accuracy profile on S_4 was not considered at that time. Let us assume that the prediction accuracy profile was as follows: 1 1 1 1 for S_1^{22}, 0 0 1 1 1 1 1 1 for S_2^{19}, and 0 1 0 1 1 1 1 1 0 1 1 1 for S_3^{23}. According to Table 2 in

the main article, the profile can be described as Finite1 for S_1, Finite2 for S_2, and Finite2 for S_3. Because the individual correctly responded to S_1 and S_2, and showed the prediction accuracy profile for S_3 that corresponds to having knowledge of S_2, but not of a deeper level, the individual's grammar is classified as $G_2 = \{S \rightarrow 1\ 2\ 3\ 4,\ S \rightarrow 1\ 1\ 2\ 3\ 4\ 2\ 3\ 4\}$. Note that we use FiniteN to refer to a sentence-level profile of prediction accuracy motivated by symbolic rules and use G_N to refer to a bearing point grammar underlying the vector of prediction accuracy profiles, in this case, (Finite1, Finite2, Finite2).

Consider another case where the prediction accuracy profile for S_3^{23} is 0 1 0 1 1 1 1 1 1 1 1 1 while the other profiles are the same as in the previous example. In this case, the profile is consistent with Finite3 for S_3 so the vector of prediction accuracy profiles is (Finite1, Finite2, Finite3). If the S_3 under consideration is the first instance of S_3, the grammar is taken to be G_R based on the participant's successful spontaneous generalization. On the other hand, if the S_3 under consideration is not the first instance of S_3 and the participant's profile of prediction accuracy for the first instance of S_3 was not classified into Finite3, the grammar is taken to be G_3 consisting of three rules: $S \rightarrow 1\ 2\ 3\ 4$, $S \rightarrow 1\ 1\ 2\ 3\ 4\ 2\ 3\ 4$, and $S \rightarrow 1\ 1\ 1\ 2\ 3\ 4\ 2\ 3\ 4\ 2\ 3\ 4$ because there is no evidence of generalization beyond experience.

3 SUPPLEMENTARY FIGURES

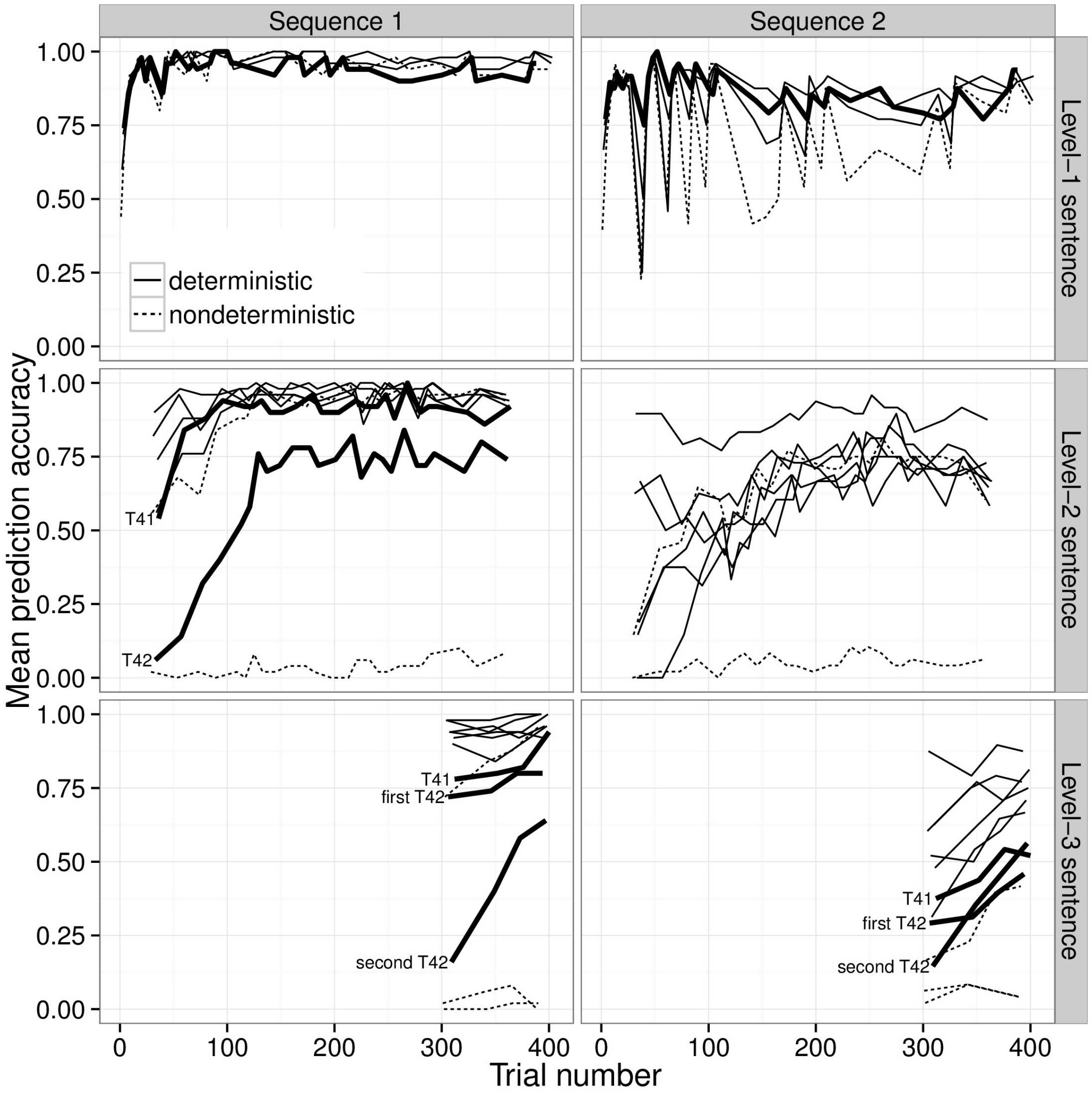

Supplementary Figure 1. Trial-level mean prediction accuracy in Experiment 1. The dashed and solid lines present the average prediction accuracies on nondeterministic (T_{11} and T_{12}) and deterministic transitions in each sentence type. The thick lines present the average prediction accuracies on T_{41} and T_{42}. Recall that different level-2 sentences (S_2 and S_2^*) were used in Sequence 1 and 2. There were no T_{41} and T_{42} in the level-2 sentence (S_2^*) used in Sequence 2.

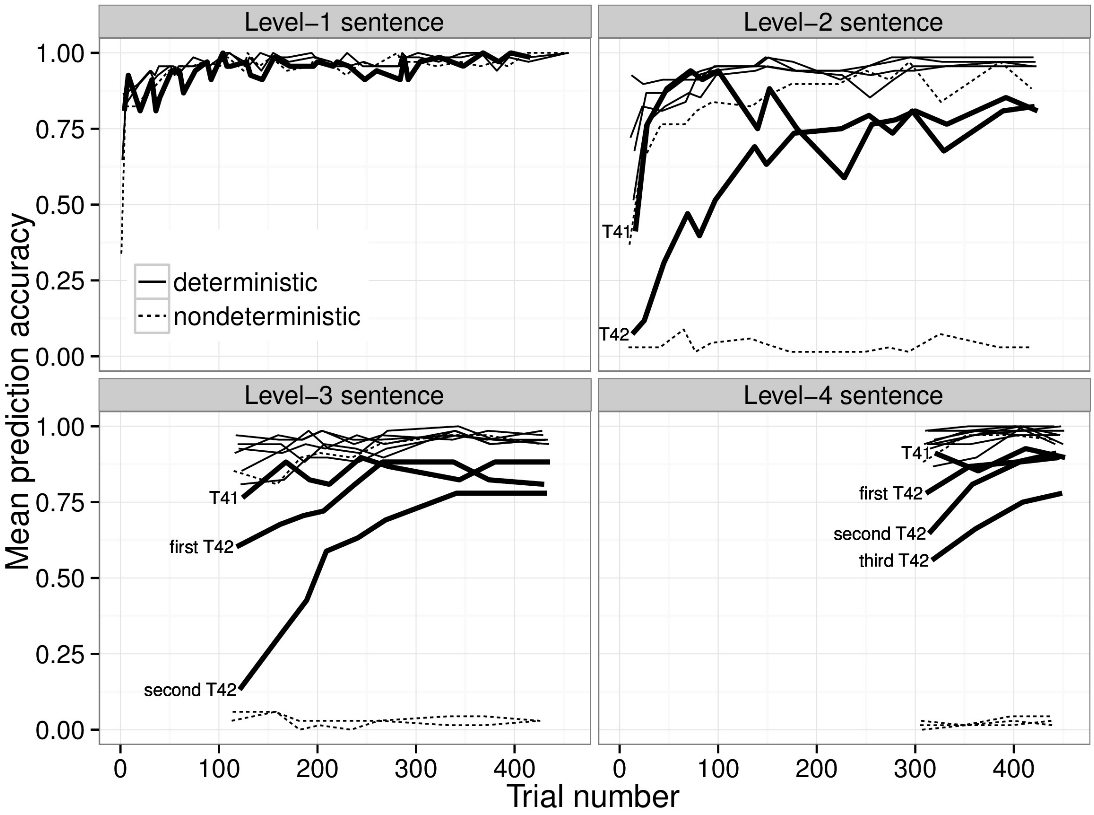

Supplementary Figure 2. Trial-level mean prediction accuracy in Experiment 2. The dashed and solid lines present the average prediction accuracies on nondeterministic (T_{11} and T_{12}) and deterministic transitions in each sentence type. The thick lines present the average prediction accuracies on the critical deterministic transitions (T_{41} and T_{42}) in each sentence type.
